# *Acthi*, a thiazole biosynthesis enzyme, is essential for thiamine biosynthesis and CPC production in *Acremonium chrysogenum*

**DOI:** 10.1186/s12934-015-0235-3

**Published:** 2015-04-11

**Authors:** Yan Liu, Wei Zhang, Liping Xie, Hong Liu, Guihua Gong, Baoquan Zhu, Youjia Hu

**Affiliations:** China State Institute of Pharmaceutical Industry, Zhangjiang Institute, 1599 Zhangheng Road, Shanghai, 201203 China; Shanghai Institute of Pharmaceutical Industry, 1320 Beijing Road (W), Shanghai, 200040 China; Present address: Luye Pharma Group Ltd., Yantai, Shandong 264003 China

**Keywords:** *Acthi*, Thiamine biosynthesis, Cephalosporin C, *Acremonium chrysogenum*, Comparative proteomics, Molecular breeding

## Abstract

**Background:**

The filamentous fungus *Acremonium chrysogenum* is an important industrial fungus and is used in the production of the β-lactam antibiotic cephalosporin C. Little is known regarding the molecular and biological mechanisms of how this industrial strain was improved by mutagenesis and molecular breeding. Comparative proteomics is one of the most powerful methods to evaluate the influence of gene expression on metabolite production.

**Results:**

In this study, we used comparative proteomics to investigate the molecular mechanisms involved in the biosynthesis of cephalosporin C between a high-producer (HY) strain and a wide-type (WT) strain. We found that the expression levels of thiamine biosynthesis-related enzymes, including the thiazole biosynthesis enzyme (*Acthi*), pyruvate oxidase, flavin adenine dinucleotide (FAD)-dependent oxidoreductase and sulfur carrier protein-thiS, were up-regulated in the HY strain. An *Acthi*-silencing mutant of the WT strain grew poorly on chemically defined medium (MMC) in the absence of thiamine, and its growth was recovered on MMC medium supplemented with thiamine. The intracellular thiamine content was changed in the *Acthi* silencing or over-expression mutants. In addition, we demonstrated that the manipulation of the *Acthi* gene can affect the hyphal growth of *Acremonium chrysogenum*, the transcription levels of cephalosporin C biosynthetic genes, the quantification levels of precursor amino acids for cephalosporin C synthesis and the expression levels of thiamine diphosphate-dependent enzymes. Over-expression of *Acthi* can significantly increase the cephalosporin C yield in both the WT strain and the HY mutant strain.

**Conclusions:**

Using comparative proteomics, four differently expressed proteins were exploited, whose functions may be involved in thiamine diphosphate metabolism. Among these proteins, the thiazole biosynthesis enzyme (ActhiS) may play an important role in cephalosporin C biosynthesis. Our studies suggested that *Acthi* might be involved in the transcriptional regulation of cephalosporin C biosynthesis. Therefore, the thiamine metabolic pathway could be a potential target for the molecular breeding of this cephalosporin C producer for industrial applications.

## Background

The β-lactam antibiotics are widely used in the treatment of infectious diseases. Cephalosporin C (CPC) is an important intermediate and is produced by the fermentation of the filamentous fungus *Acremonium chrysogenum*. This fungus, first isolated in 1948 from Sardinian coastal seawater, produces an antibiotic against gram-positive and gram-negative bacteria [1]. To date, the CPC yield has been increased to industrial production levels by recursive mutagenesis [2]. In addition to traditional strain improvement methods, recombinant DNA technology has been applied to *A. chrysogenum* to improve CPC production [3].

CPC biosynthesis and transportation and their regulation patterns have been identified, and considerable progress has been made in understanding CPC biosynthesis in *A. chrysogenum* [4-8]. In this fungus, global regulators, such as *AcVEA*, *CPCR1*, *CRE1* and *PACC,* regulate the expression of CPC biosynthesis genes and CPC production [6,9-11]. In filamentous fungi, secondary metabolism and morphogenesis are tightly connected. The hyphal differentiation phase and arthrospore formation coincide with the maximum rate of β-lactam antibiotic synthesis. Arthrospores can enhance the synthesis of β-lactam antibiotic and appear to be a determining factor in high-yield strains [12]. *AcVEA*, *CPCR1* and *AcsepH* are involved in cellular differentiation and arthrospore formation in *A. chrysogenum*, both of which affect CPC biosynthesis [4,6,13].

Despite the considerable progress that has been made in understanding CPC biosynthesis in *A. chrysogenum*, including the decoding of the complete nucleotide sequence of the 27,266 bp mitochondrial genome of *A. chrysogenum* recently [14], the exploitation of *A. chrysogenum* requires knowledge from other -omics areas of study, such as proteomics. Proteomics is one of the most powerful methods to evaluate the influence of gene expression on metabolite production. This technique has been successfully used to study other ascomycete fungi, such as *Penicillium chrysogenum* [15] and *Aspergillus fumigatus* [16]. Jami et al. compared the proteomic profiles of a wild-type strain and industrial strains of the filamentous fungus *Penicillium chrysogenum* and identified a series of differentially expressed proteins, including penicillin biosynthetic enzymes and other secondary metabolic proteins and carbohydrate metabolism- and energy-related proteins. Comparative proteomic approaches can be used to explore proteins whose functions are still unclear but that may play important roles in CPC biosynthesis.

**Figure 1:**
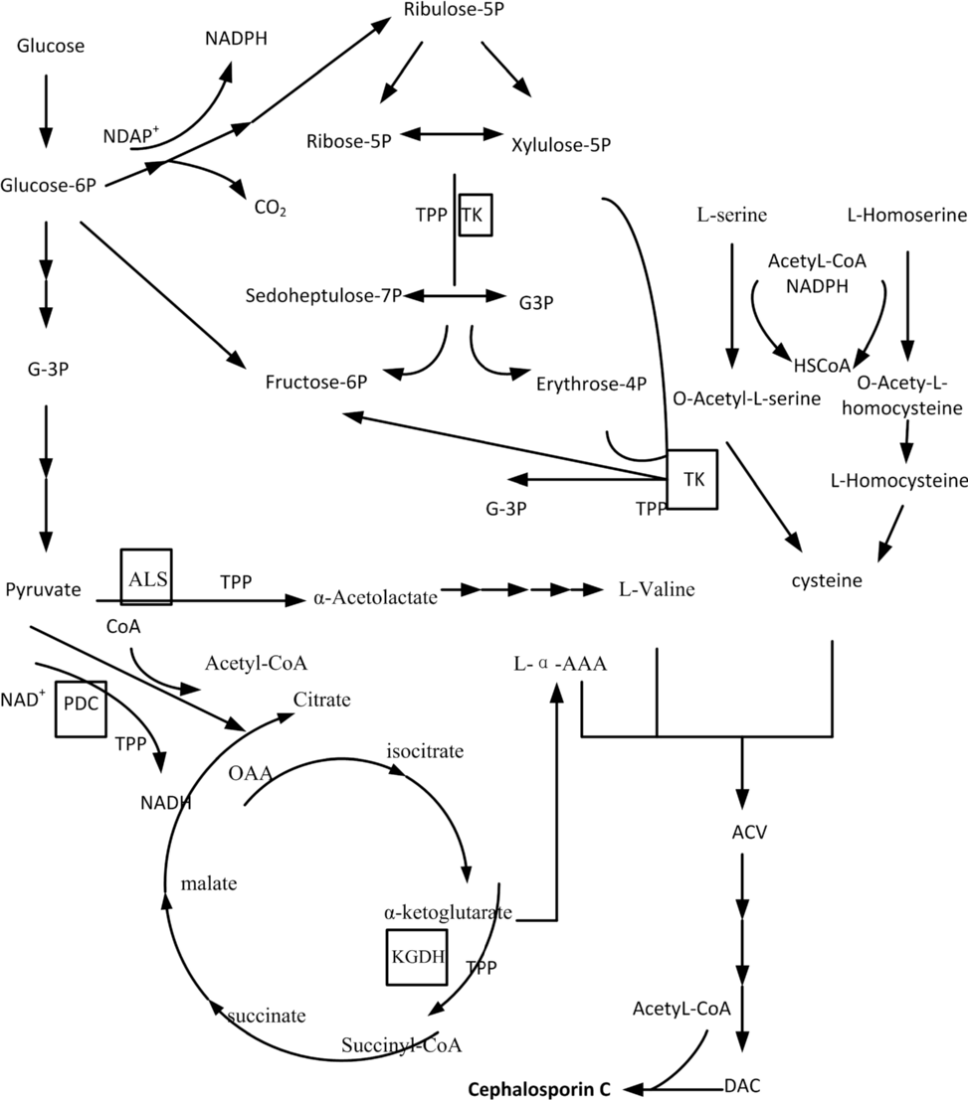
Proposed roles of TPP and TPP-dependent enzymes in CPC biosynthesis in *Acremonium chrysogenum*. TPP: thiamine diphosphate, a biologically active form of thiamine. ALS (acetolactate synthase), TK (transketolase), PDC (pyruvate decarboxylase) and KGDH (α-ketoglutarate dehydrogenase) are TPP-dependent enzymes (square open boxes). ALS is involved in L-valine synthesis; PDC decarboxylates pyruvate to generate acetyl-CoA. Most NADPH is generated in the pentose phosphate pathway, which is related to TK activity. Acetyl-CoA and NADPH are important in cysteine biosynthesis. L-valine, cysteine, and L-α-AAA are the precursor acids in CPC biosynthesis; thus, acetyl-CoA and NADPH are also important in CPC biosynthesis.

Thiamine is an essential cofactor in carbohydrate metabolism and is involved in the processes of glycolysis, the citric acid cycle and the pentose-phosphate cycle (Figure 1) [17,18]. Thiamine is the precursor of thiamine diphosphate, which is a cofactor for over 20 well-characterized enzymes, including pyruvate decarboxylase (PDC), pyruvate dehydrogenase (PDH), α-ketoglutarate dehydrogenase (KGDH), branched-chain α-ketoacid dehydrogenase (BKDH), transketolase (TK) and acetolactate synthase (ALS) [19]. These enzymes are involved in cell bioenergetics and ATP and NADPH synthesis. Moreover, these enzymes participate in the biosynthesis of pentose (required for nucleotide synthesis), amino acids and other organic compounds important in cell metabolism. Thiamine biosynthesis involves two independent pathways: synthesis of the thiazole and the production of the pyrimidine moieties of thiamine, which are then coupled to form thiamine [20,21]. Microorganisms, fungi and plants can synthesize thiamine, but animals and humans must obtain thiamine from their food. The biosynthesis of the thiazole moiety in prokaryotes has been studied extensively. It involves a complex oxidative condensation of 1-deoxy-D-xylulose-5-phosphate, glycine or tyrosine and cysteine, catalyzed by five different enzymes throughout the pathway [22].

In eukaryotes, the biosynthesis of thiazole has not yet been well described, with only limited studies published to date [23-25]. The identified thiazole biosynthesis family in eukaryotes includes *Thi4p* from *Saccharomyces cerevisiae* [26], *Thi1p* from *Arabidopsis thaliana* [27], *CyPBP37* from *Neurospora crassa* [28,29], and *ThiA* from *Aspergillus oryzae* [30,31]*.* These genes are highly expressed in yeasts and filamentous fungi and are subject to transcriptional suppression by thiamine [26,28,32]. These enzymes are involved in the biosynthesis of pentose, amino acids and other organic compounds of cell metabolism. The pathways in which they are involved may thus affect CPC biosynthesis in *A. chrysogenum* via carbohydrate metabolism and energy, CPC biosynthetic precursors, cellular differentiation and arthrospore formation.

In this study, we analyzed the comparative proteomics between a high-yield (HY) (*A. chrysogenum* 84-3-81-41) CPC producer and a wild-type (WT) (*A. chrysogenum* ATCC11550) strain using two-dimensional gel electrophoresis (2-DE). Proteins of interest were identified by mass spectrometry (MS). The ActhiS level (thiazole biosynthesis enzyme) was determined to be positively correlated with CPC production. We then further characterized the role of the *Acthi* gene. Based on the results of ActhiS silencing and over-expression in *A. chrysogenum*, we postulated that ActhiS plays an important role in CPC biosynthesis. The thiamine biosynthesis pathway is highly correlated with the expression level of CPC biosynthesis genes, the production of arthrospores and precursor amino acids for CPC biosynthesis. Thiamine biosynthesis, particularly ActhiS, may be a potential manipulating target for the molecular breeding of CPC producers.

## Results

### Comparative intracellular proteomic analysis of the HY and WT strains

**Figure 2:**
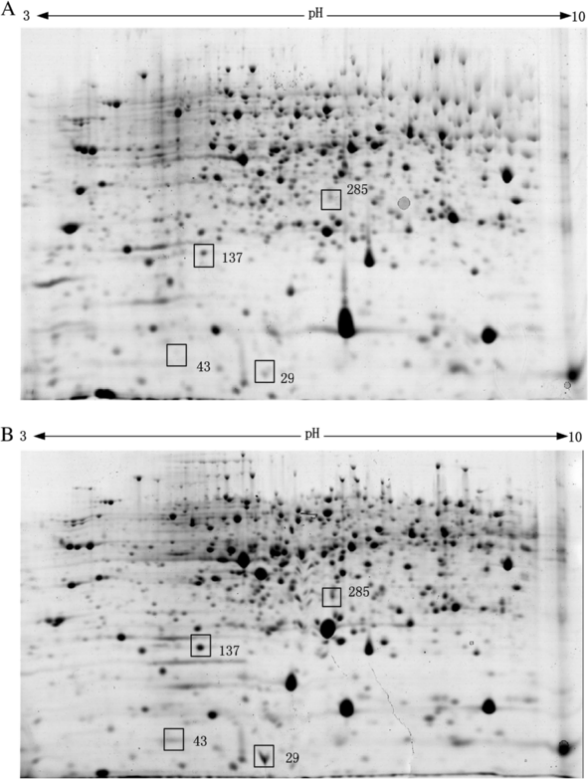
2D-PAGE analyses of the intracellular proteomes of the HY and WT strains. Details of the sample preparation and 2D gel-electrophoresis are described in the Methods. The identities of the protein spots were determined by MALDI-TOF MS analysis. The spots showed differentially expressed proteins whose intensities were at least 2-fold higher or lower at a statistical significance level (p < 0.05, Student’s t-test). (**A**) A 2-D gel of the intracellular proteome of WT strains after a 7-day fermentation. (**B**) A 2-D gel of the intracellular proteome of HY strains after a 7-day fermentation.

**Figure 3:**
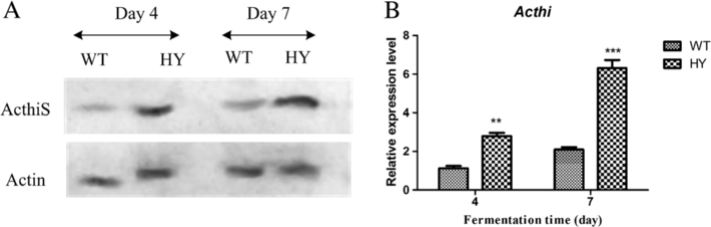
Translational and transcriptional analysis of the *Acthi* gene in the HY and WT strains. (**A**) Western blotting analysis of ActhiS in the WT and HY strains. The protein samples were extracted on fermentation days 4 and 7. Fifteen micrograms of total protein was loaded for Western blotting analysis with anti-ActhiS antibodies. *Actin* was the loading control. ActhiS expression was higher in the HY strain compared to the WT strain. (**B**) The relative expression of *Acthi* in WT and HY strains. The RNA samples were extracted on fermentation days 4 and 7 and were subjected to RT-qPCR with *Acthi* and *actin*-specific primers, as described in the Methods. The relative mRNA abundance levels were calculated against *actin* gene levels. The histogram amplitude represents the average of three experiments. Error bars represent the standard deviations. A significant difference in the relative expression level of the *Acthi* gene in HY compared with that in WT is indicated as “*”; p value confidence levels: “**” < 0.01 and “***” < 0.001. *Acthi* was over-expressed in the HY stain throughout the fermentation process.

To investigate the total protein expression profiles of the HY and WT strains, a comparative proteomics approach, involving 2-DE followed by an assessment of the characteristics of the isolated proteins by tandem MS (MS/MS), was used. The 2-DE analyses of the intracellular proteins were performed in triplicate to allow statistical analysis, and Student’s t-test was used to determine if the relative changes in the protein expression levels were statistically significant. The 2-DE gels for the HY and WT strains are shown in Figure 2. The isoelectric points of the spots ranged from pH values of 3 to pH values of 10 (non-linear). This experiment was performed in triplicate and showed highly reproducible results. More than 1900 intracellular proteins were obtained on the 2-D gels, and 37 differentially expressed spots were identified. These spots were further analyzed by matrix-assisted laser desorption/ionization MS (MALDI-MS) and liquid chromatography MS/MS (LC-MS/MS). A total of 26 spots were characterized, and four identified proteins, including pyruvate oxidase, the flavin adenine dinucleotide (FAD)-dependent oxidoreductase superfamily, sulfur carrier protein-thiS and thiazole biosynthetic enzyme, were related to thiamine metabolism (Table 1). The thiazole biosynthetic enzyme of thiamine biosynthesis was highly expressed in the HY strain. Putative pyruvate oxidase and putative FAD-dependent oxidoreductase superfamily were involved in thiamine diphosphate (TPP) and FAD biosynthesis and were also highly expressed in the HY strains compared to the WT strains. Thiamine is an essential cofactor in *A. chrysogenum*, which might affect CPC production via carbohydrate metabolism and energy and amino acid biosynthesis. The DNA sequence of the thiazole biosynthetic enzyme was obtained from our own RNA-seq results and has been deposited into a public database [GenBank: KF010923]. However, the coding sequences of pyruvate oxidase and the putative FAD-dependent oxidoreductase superfamily of *A. chrysogenum* were not determined in our own RNA-seq analysis of *A. chrysogenum*. To test whether the expression of the *Acthi* gene was different in the HY and WT strains, Western blotting and RT-qPCR experiments were conducted using protein and RNA extracted from the samples on fermentation days 4 and 7. The results are shown in Figure 3. The western blotting results demonstrated that ActhiS expression is higher in the HY strain than in the WT strain (Figure 3A); RT-qPCR results corresponded with the Western blotting and comparative proteomic analysis (Figure 3B).

**Table 1:** **Identification of differentially expressed proteins involved in thiamine metabolism between the HY and WT strains during fermentation**

**Spot ID**	**Accession No.**	**Homologous protein name**	**Species**	**MV/pI est**	**MV/pI theo**	**MOWSE score**	**Pep-count**	**Fold change HY/WT**
137	gi|260560941	pyruvate oxidase	*Lactobacillus jensenii SJ-7A-US*	22.26/5.2	66.99/4.9	93	18	2.9
29	gi|242767495	FAD-dependent oxidoreductase superfamily	*Talaromyces stipitatus* ATCC 10500	11.43/6.2	13.6/6.26	70	18	2.32
43	gi|289580250	sulfur carrier protein-thiS	*Natrialba magadii* ATCC 43099	11.05/4.12	9.5/4.48	92	7	−2.0
285	gi|532596872	thiazole biosynthetic enzyme	*Acremonium chrysogenum ATCC* 11550	33.38/6.0	34.44/6.01	280	7	2.1

### Over-expression and silencing of *Acthi* in WT *A. chrysogenum*

**Figure 4:**
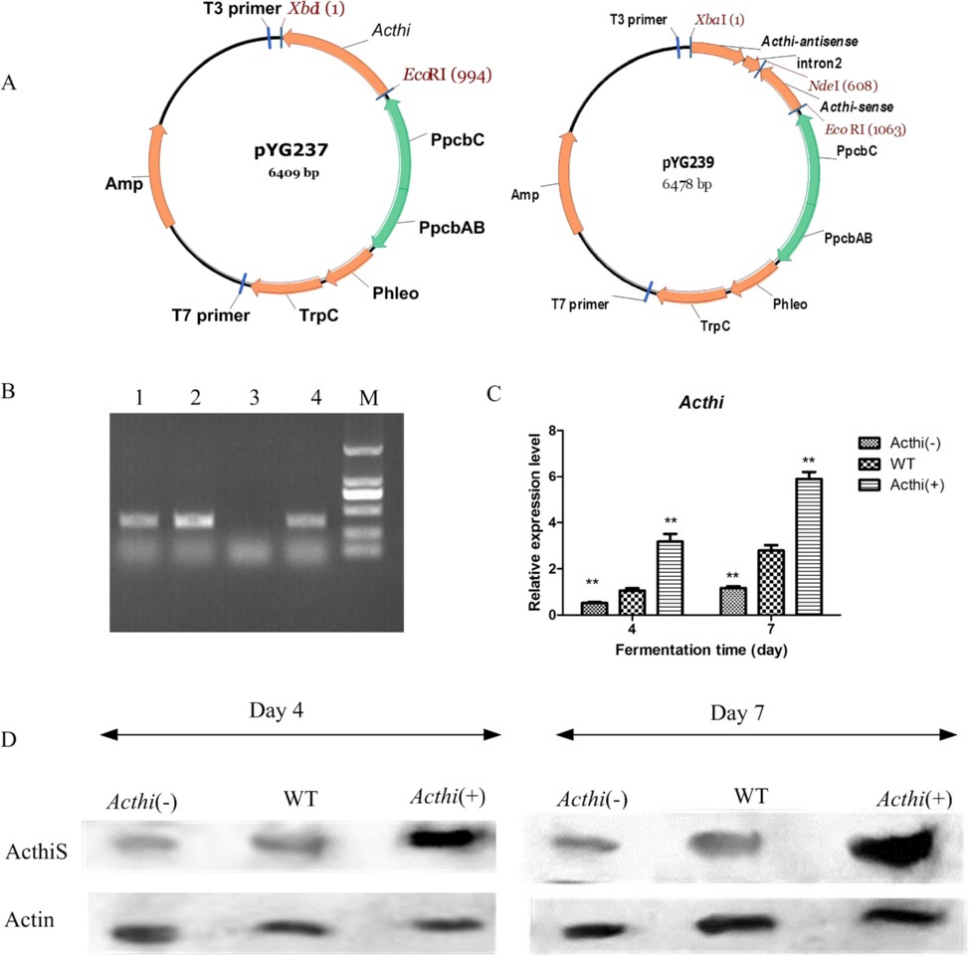
Plasmids used for the construction *Acthi* mutants in *A. chrysogenum* and in the verification of *Acthi* expression. (**A**) *Acthi* over-expression and silencing vector. pYG237: *Acthi* gene over-expression plasmid; pYG239: *Acthi* gene silencing plasmid. (**B**) PCR verification of pYG237- and pYG239-transformed *A. chrysogenum*. Lane 1: *Acthi*(−), lane 2: *Acthi*(+), lane 3: WT, lane 4: positive control; M: DL2000 DNA marker. The *phleo* gene was detected in the *Acthi* mutants. (**C**) *Acthi* transcription levels in *Acthi* mutants. Relative mRNA abundance levels were standardized against *actin* gene levels. Error bars represent the standard deviations from three independent experiments. Significant differences of the relative *Acthi* gene expression levels in *Acthi*(−) and *Acthi*(+) compared with the WT are indicated as “*”; p value confidence level: “**” < 0.01. The transcription levels of *Acthi* were higher in the *Acthi*(+) strain and lower in the *Acthi*(−) strain compared with the WT strain. (**D**) ActhiS expression levels in *Acthi* mutants. Fifteen micrograms of total protein from *Acthi*(−), WT and *Acthi*(+) were loaded for Western blotting analysis with anti-ActhiS antibodies. *Actin* was the loading control. ActhiS expression showed a similar trend in the *Acthi* mutants.

To investigate the function of *Acthi*, pYG237 and pYG239 (for *Acthi* over-expression and silencing, respectively, Figure 4A) were transformed into WT *A. chrysogenum*. The *Acthi* over-expression transformants and the *Acthi* silencing mutants were selected with 5 μg mL^−1^ phleomycin and were verified by PCR amplification of the *phleo* gene (Figure 4B). More than 20 transformants were selected, and the most representative *Acthi*(+) (*Acthi* over-expression mutant) and *Acthi*(−) (*Acthi* silencing mutant) strains were used for the subsequent experiments. The RT-qPCR and Western blotting experiments were conducted to investigate the expression of the *Acthi* gene in *Acthi*(−), WT and *Acthi*(+) strains (Figure 4C and 4D). The RT-qPCR results demonstrated that *Acthi* transcription was higher in the *Acthi*(+) strain and lower in the *Acthi*(−) strain compared with the WT strain. Western blotting returned similar results.

### The role of *Acthi* in thiamine biosynthesis

**Figure 5:**
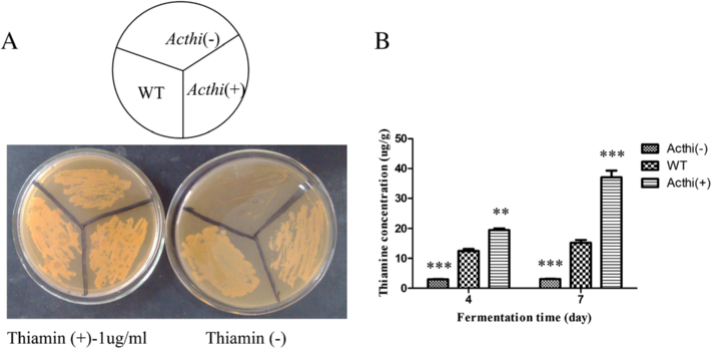
Role of *Acthi* in thiamine biosynthesis. (**A**) Growth of the *Acthi* mutants on MMC medium. *Acthi*(−), WT and *Acthi*(+) were inoculated on MMC medium in the presence or absence of thiamine. Plates were incubated at 28°C for 7 days. The *Acthi* silencing mutant grew poorly; however, rapid growth was recovered in thiamine-supplemented medium. (**B**) Detection of thiamine content in the *Acthi* mutants. The mycelium of *Acthi* mutants was collected on fermentation days 4 and 7. Samples were treated as described in the Methods and then detected by a microplate reader. Data were presented as the means ± SD with n = 3 independent assays per data point. Significant differences in the thiamine contents in *Acthi*(−) and *Acthi*(+) compared with WT are indicated as “*”; p value confidence levels: “**” < 0.01 and “***” < 0.001. The thiamine content in *Acthi*(+) reached approximately 38 μg g^−1^ by fermentation day 7, which was significantly higher than in the WT strain. In contrast, the thiamine content was decreased in the *Acthi*(−) strain.

Previous studies have indicated that the yeast *Thi4p* is a thiamine biosynthetic enzyme [33]. To test whether *Acthi* from *A. chrysogenum* is an orthologous gene, *Acthi*(−), WT and *Acthi*(+) were inoculated on MMC lacking thiamine or supplemented with 1 μg mL^−1^ thiamine. WT and *Acthi*(+) can grow in the absence of thiamine, whereas the *Acthi*-silenced mutant grew poorly (Figure 5A). In contrast, all transformants grew normally on thiamine-supplemented medium.

In addition, we examined the intracellular thiamine content of *Acthi*(−), WT and *Acthi*(+) on fermentation days 4 and day 7. First, 0.1-1 μg mL^−1^ of a standard thiamine working solution was mixed with alkaline potassium ferricyanide, which gave us the thiamine standard curve (y = 5129.7x-4957, R^2^ = 0.9949). As expected, the levels of thiamine in *Acthi*(−) were lower, whereas those in *Acthi*(+) were higher (Figure 5B). These results indicated that *Acthi* encodes a thiazole enzyme. The silencing of *Acthi* blocked thiamine biosynthesis, while *Acthi* over-expression can stimulate thiamine production.

### *Acthi* is involved in hyphal growth and spore germination in *A. chrysogenum*

**Figure 6:**
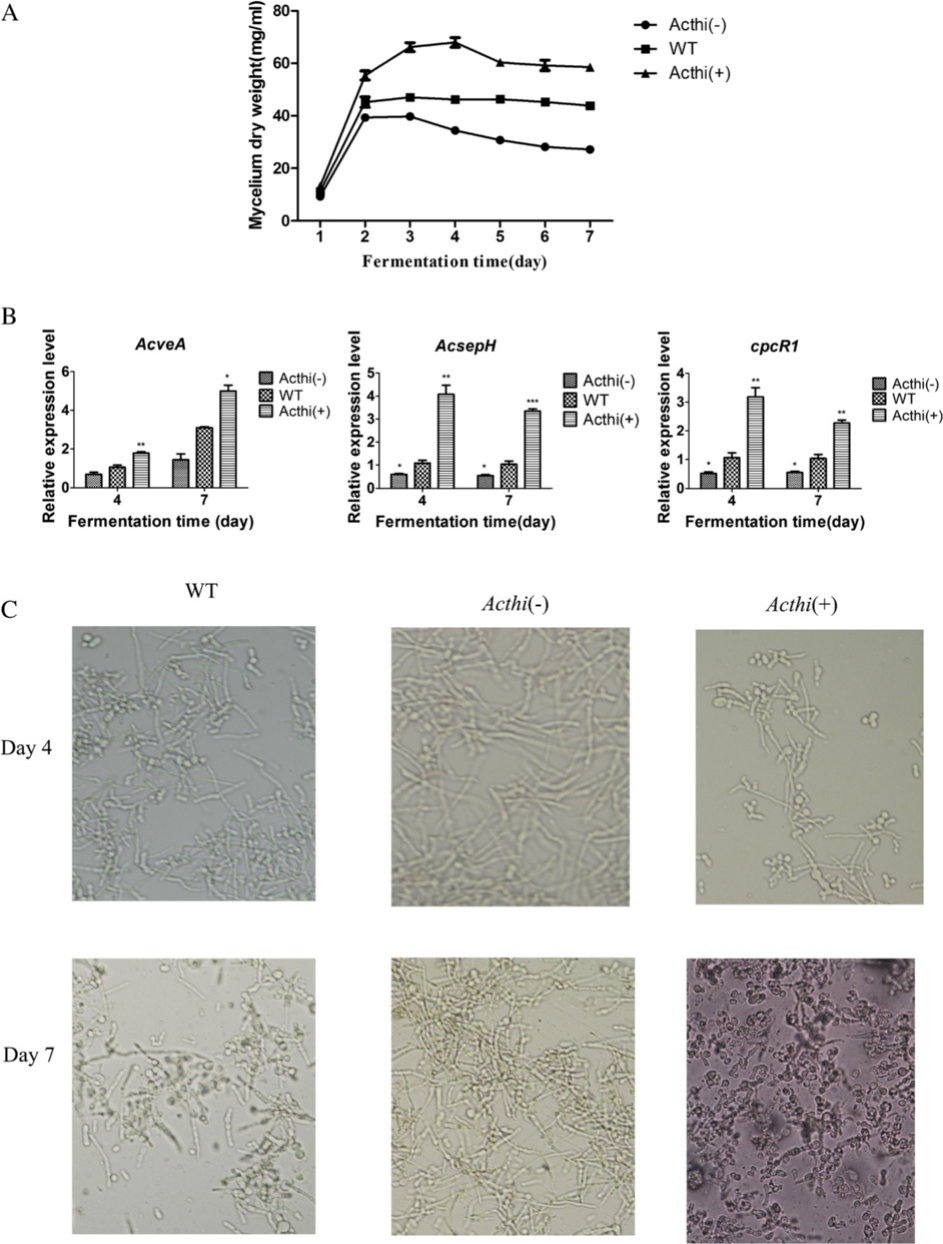
*Acthi* is involved in hyphal growth and spore germination in *A. chrysogenum.* (**A**) The biomass levels of WT, *Acthi*(−) and *Acthi*(+) on fermentation days 4 and 7. The biomass of *Acthi*(+) dramatically increased in the fermentation phase compared with the WT strain, and the mycelium weight of the *Acthi* (−) strain was approximately one-half that of the WT strain. (**B**) Relative expression levels of *AcevA*, *AcsepH*, *cpcR1* in WT, *Acthi*(−) and *Acthi*(+). The primers used for RT-qPCR are listed in Table 2. The relative mRNA abundance levels were standardized against the *actin* gene levels. Error bars represent the standard deviations from three independent experiments. Significant differences in the relative expression levels for *AcevA*, *AcsepH*, *cpcR1* in *Acthi*(−) and *Acthi*(+) compared with WT are indicated as “*”; p value confidence levels: “*” < 0.05, “**” < 0.01, and “***” < 0.001. The transcription levels of *AcveA*, *AcsepH*, *cpcR1* showed similarly higher levels in *Acthi*(+) and lower levels in *Acthi*(−) compared to those in WT. (**C**) Morphological characteristics of WT, *Acthi*(−) and *Acthi*(+) on fermentation days 4 and 7. The mycelium growth of WT is different from that of the *Acthi* mutants.

The mycelium biomass of the *Acthi*(−) strain after 7 days of fermentation was approximately one-half that of the WT strain, and the mycelium weight of the *Acthi* (+) strain was increased by 50% compared to the WT strain (Figure 6A). To further explore this finding, the transcription of hyphal growth and spore germination-related genes (*AcveA*, *AcsepH*, *cpcR1*) in *Acthi*(−), WT and *Acthi*(+) were measured by RT-qPCR (Figure 6B). The transcriptional level of *AcveA*, which is involved in cellular differentiation and arthrospore formation, indicated that *AcveA* transcription in *Acthi*(−) was lower than in WT and higher in *Acthi*(+). Similarly, the expression levels of *AcsepH* (related to the fungal physiological state) and *cpcR1* (the arthrosporulation-control gene) in the *Acthi*(−) were lower than in the WT strain and higher in *Acthi*(+). Aerial hyphal growth and spore germination of the WT and *Acthi* mutants were observed at different fermentation stages by microscopy and image analysis (Figure 6C). Compared to the WT strain, the mycelium growth of the *Acthi*(−) strain displayed an obvious delay on fermentation day 4; however, conidia can be seen in *Acthi*(+) at this time point. On day 7, the conidia number was lower in the *Acthi*(−) strain than in the WT strain, while the *Acthi*(+) strain produced a large number of conidia. These data indicate that the *Acthi* gene may be related to the cellular differentiation and arthrospore formation of *A. chrysogenum*.

### The silencing and over-expression of *Acthi* affected cephalosporin C production by altering the expression of cephalosporin biosynthetic genes in *A. chrysogenum* during fermentation

**Figure 7:**
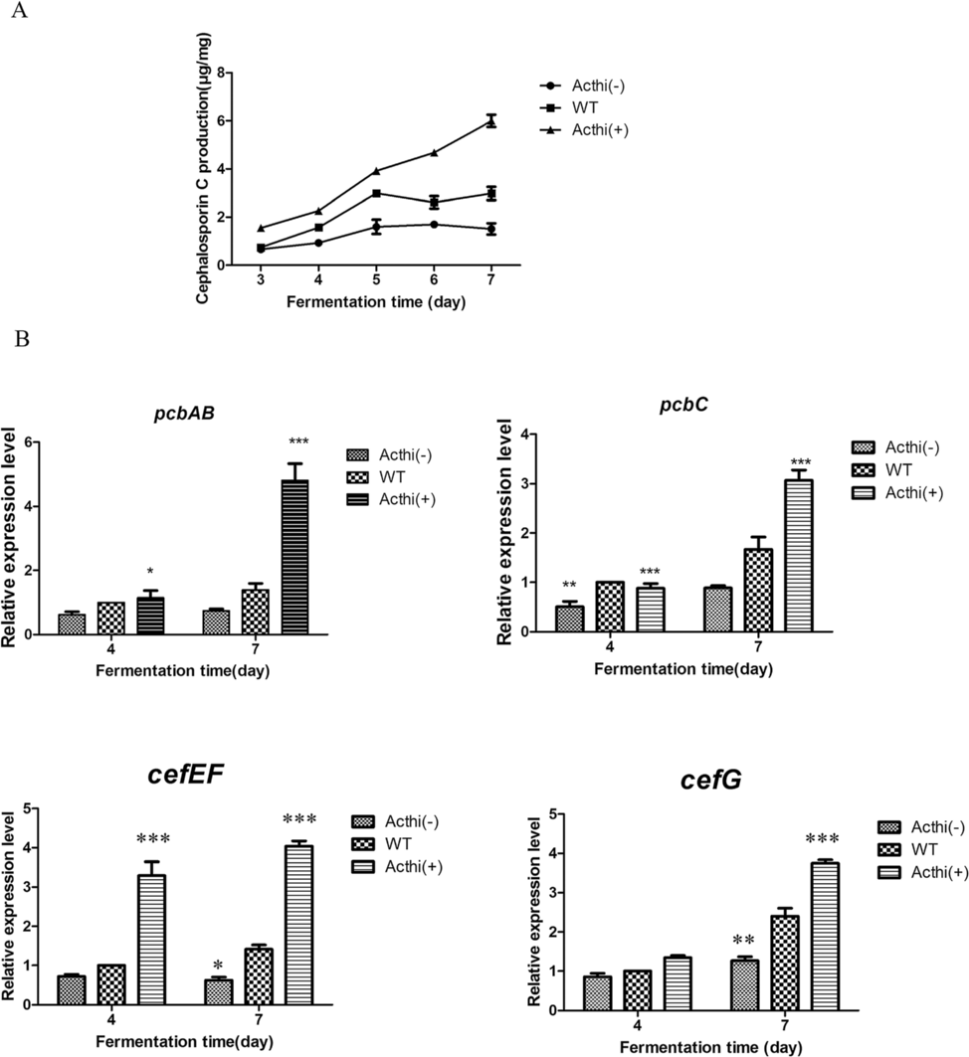
Comparison of CPC production and transcriptional analysis of CPC biosynthetic genes in WT, *Acthi*(−) and *Acthi*(+). (**A**) CPC production of WT, *Acthi*(−) and *Acthi*(+). Error bars represent the standard deviations from three independent experiments. The procedures of fermentation and the detection CPC production were performed as described in the Methods. The CPC production of *Acthi*(+) was dramatically increased and had a 42.4% decrease in *Acthi*(−) compared with WT. (**B**) The expression of cephalosporin biosynthetic genes in WT, *Acthi*(−) and *Acthi*(+). The primers used for RT-qPCR are listed in Table 2. The relative abundance of the mRNAs was normalized against the *actin* gene levels. Error bars represent the standard deviations from three independent experiments. Significant differences in the relative expression levels of the cephalosporin biosynthetic genes in *Acthi*(−) and *Acthi*(+) compared with WT are indicated as “*”; p value confidence levels: “*” < 0.05, “**” < 0.01, and “***” < 0.001.

In filamentous fungi, arthrospores enhanced the synthesis of the β-lactam antibiotic and appeared to be a determining factor in high-yielding strains [12]. As described above, we have demonstrated that *Acthi* expression levels can affect hyphal growth and spore germination. To determine the relationship between the *Acthi* gene and CPC production, we studied the CPC production of the WT, *Acthi*(−) and *Acthi*(+) strains. In comparison with the WT strain, the CPC yield was decreased by 42.4% in the *Acthi*(−) strain and was increased significantly by 68.7% in the *Acthi*(+) strain (Figure 7A). The CPC production/mycelium dry weight showed a similar trend. These results indicated that *Acthi* plays a key role during CPC fermentation and that *Acthi* over-expression can enhance CPC production, as seen in the *Acthi*(+) strain.

We also examined the transcriptional levels of the CPC biosynthetic genes *pcbAB* (L-α-aminodipyl-L-cysteinyl-D-valine synthetase gene), *pcbC* (isopenicillin N-synthetase gene), *cefEF* (bifunctional deacetoxycephalosporin C synthase/hydroxylase gene), and *cef* (DAC acetyltranferase gene) in the WT and mutant strains (Figure 7B). Consistent with the CPC production, the transcriptional levels of *pcbAB*, *pcbC*, *cefG* and *cefEF* were significantly down- and up-regulated in the *Acthi*(−) and *Acthi*(+) strains, respectively. These results showed that the silencing and over-expression of the *Acthi* gene in *A. chrysogenum* altered the CPC biosynthesis during fermentation. These findings also suggest that *Acthi* plays a role in regulating the transcription of CPC biosynthesis genes.

### Detection of precursor amino acids for CPC synthesis in *A. chrysogenum* mutants

**Figure 8:**
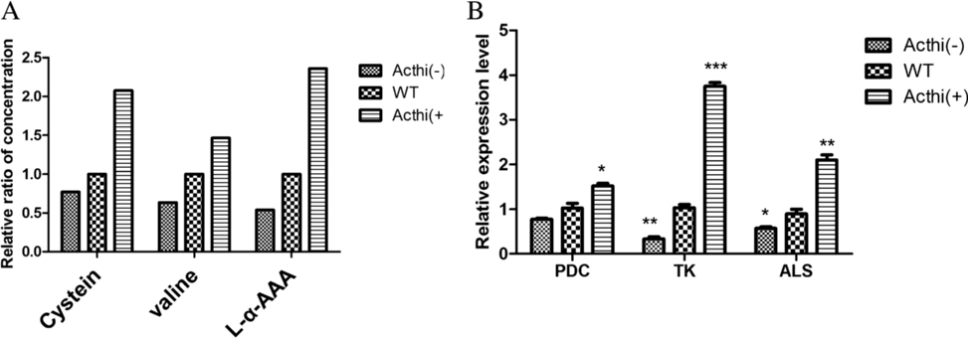
Effects of the *Acthi* gene on precursor amino acids and TPP-dependent enzymes in *WT, Acthi*(−) *and Acthi*(+). (**A**) Relative quantification of intracellular precursor amino acids (cysteine, valine and L-α-AAA) for CPC synthesis in *A. chrysogenum* mutants. Details of the sample preparation and GC/MS analysis for amino acids are described in the Methods. The concentrations of cysteine, valine and L-α-AAA showed similar trends in the *Acthi* mutants. (**B**) RT-qPCR analyses assessing the expression of TPP-dependent enzymes (PDC, TK and ALS) in *A. chrysogenum* mutants on fermentation day 7. The relative mRNA abundance levels were standardized against the *actin* gene levels. Error bars represent standard deviations from three independent experiments. Significant differences in the expression of TPP-dependent enzymes (PDC, TK and ALS) in *Acthi*(−) and *Acthi*(+) compared with WT are indicated as “*”; p value confidence levels: “*” < 0.05, “**” < 0.01, and “***” < 0.001. TK appeared to be most sensitive to the *Acthi* mutants. The PDC gene expression levels in *Acthi*(+) and *Acthi*(−) were not obviously significantly different. The ALS expression levels were increased by over 2-fold in *Acthi*(+) and showed a significant decline in *Acthi*(−).

To identify the relationship between the *Acthi* gene and CPC biosynthesis, we studied the intracellular contents of precursor amino acids (cysteine, valine and L-α-AAA) for CPC synthesis (Figure 8A) by gas chromatography-MS (GC-MS). The cysteine concentration in *Acthi*(+) was 2.07-fold higher than in WT, and the concentration in *Acthi*(−) declined compared to WT. L-valine is synthesized from pyruvate, which is catalyzed by a TPP-dependent acetolactate synthase. In metabolic flux analysis, the valine concentrations were increased by 1.46-fold in *Acthi*(+) and by 0.635-fold in *Acthi*(−) compared to WT. L-α-AAA showed a similar trend as cysteine and valine in the *Acthi* mutants.

### TPP-dependent enzyme expression is involved in CPC biosynthesis in *A. chrysogenum* mutants

To correlate the *Acthi* gene with CPC biosynthesis, we analyzed the expression levels of several genes encoding TPP-dependent enzymes (PDC, TK, ALS) (Figure 8B). TK appeared to be most sensitive to *Acthi* gene over-expression and silencing. In *Acthi*(+), TK expression was more than 3-fold higher, while in *Acthi*(−), less than half of the WT expression was observed. The ALS expression patterns were similar to the TK expression patterns (with an over 2-fold increase in *Acthi*(+) and a significant decline in *Acthi*(−)). However, the expression of the PDC gene in *Acthi*(+) and *Acthi*(−) was increased or decreased, respectively, compared to WT, although these differences were not significant.

### The overexpression of the *Acthi* gene can significantly increase CPC production in the HY strain

**Figure 9:**
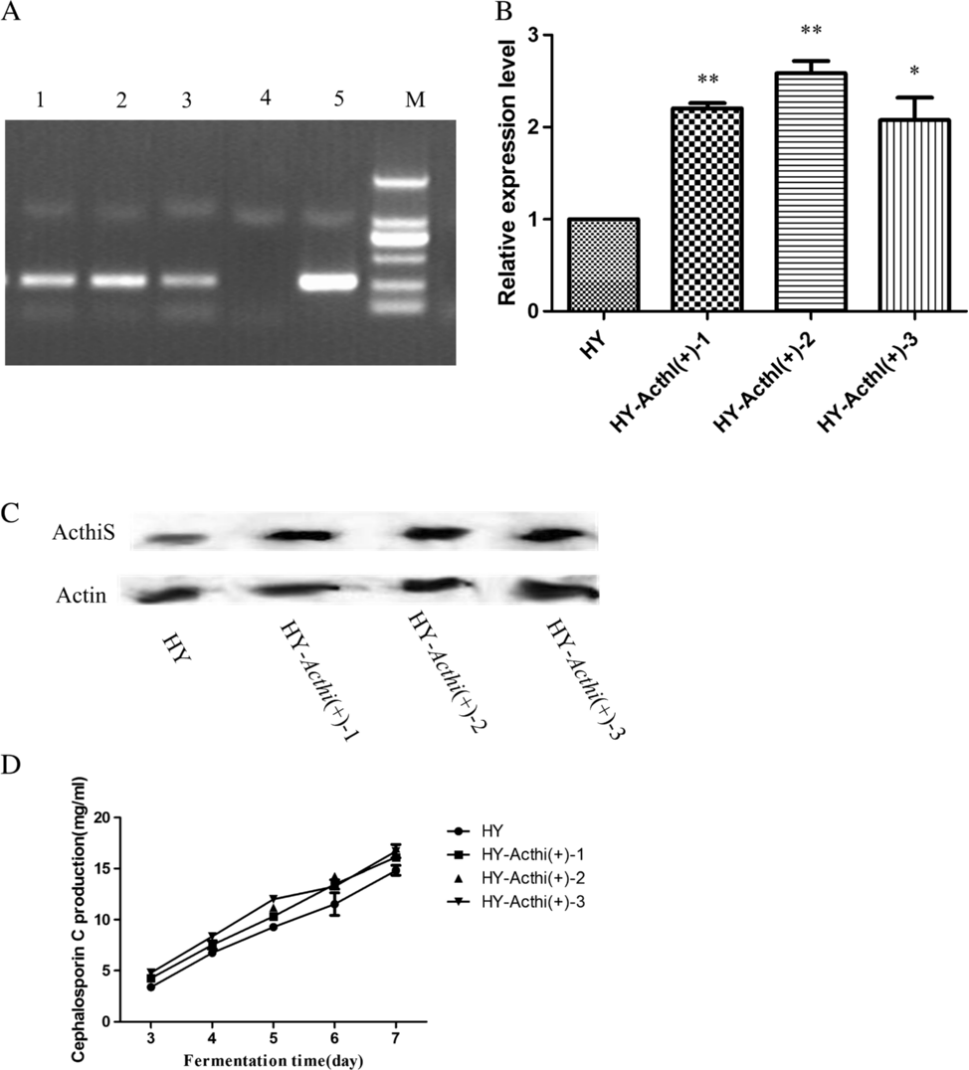
Impact of *Acthi* over-expression on CPC production in the HY strain. (**A**) PCR verification of pYG239/HY. Lanes 1, 2, and 3: genomic DNA of HY-*Acthi*(+)-1.2.3; lane 4: genomic DNA of HY; lane 5: positive control; M: DL2000 DNA Marker. The *phleo* gene was detected in the *Acthi* mutants. (**B**) *Acthi* expression levels in the HY-*Acthi*(+) transformants. The relative mRNA abundance levels were normalized against the *actin* gene levels. Error bars represent the standard deviations from three independent experiments. Significant differences in the *Acthi* expression levels in HY-*Acthi*(+) mutants compared with HY are indicated as “*”; p value confidence levels: “*” < 0.05, “**” < 0.01, and “***” < 0.001. The *Acthi* gene transcription levels in HY-*Acthi*(+)-1,2,3 were higher compared to those in the HY strain during fermentation. (**C**) Western blotting analysis of ActhiS expression in HY-*Acthi*(+) transformants. Fifteen micrograms of total protein isolated from fermented mycelia on day 7 was loaded for Western blotting analysis with anti-ActhiS antibodies. Actin was used as the loading control. The expression levels of ActhiS in HY-*Acthi*(+)-1,2,3 were higher compared to those in the HY strain during the fermentation. (**D**) Comparison of CPC production in the HY strain and in the mutants HY-*Acthi*(+)-1,HY-*Acthi*(+)-2 and HY-*Acthi*(+)-3 on fermentation day 7. Data were presented as the means ± SD (n = 3). The CPC production of the transformants was significantly improved over that of the HY stain.

According to the results of comparative proteomics and functional study of the *Acthi* gene, *Acthi* may be positively correlated with CPC production. The over-expression vector pYG239 was then introduced into the HY strain by PEG-mediated protoplast transformation. Transformants were selected by 5 μg mL^−1^ phleomycin and verified by PCR amplification of the *phleo* gene (Figure 9A). We chose three transformants (HY-*Acthi*(+)-1, HY-*Acthi*(+)-2, and HY-*Acthi*(+)-3) to perform subsequent analysis, including CPC production. Those strains were carried into fermentation along with the HY strain. Transcription and protein analyses revealed that the *Acthi* gene expression levels in HY-*Acthi*(+)-1,2, and 3 were higher than that of the HY strain during fermentation by RT-qPCR and Western blotting (Figure 9B,C). Finally, the CPC production of the mutants was significantly improved over the HY strain (Figure 9D): HY-*Acthi*(+)-1 had a 10.85% increase, HY-*Acthi*(+)-2 had an 11.44% increase, and HY-*Acthi*(+)-3 had an 11.2% increase (the CPC production/mycelium dry weights were also increased). These data suggested that *Acthi* gene over-expression can improve CPC production in the HY strain.

## Discussion

Although *A. chrysogenum* has been extensively modified by genetic manipulation and mutagenesis during the last decades to improve CPC production, its fermentation yield is still far less than that of penicillin production. Our lab has been working on *A. chrysogenum* for many years using genetic engineering technology for the molecular breeding of CPC producers and the improvement of CPC production, especially in industrial strains. In previous work, we used RNA-seq technology to analyze the *A. chrysogenum* transcriptome [34]. In this study, we used a comparative proteomics study to identify the differentially expressed proteins in an HY strain and a WT strain during fermentation. More than 1900 intracellular proteins were obtained on 2-D gels, and 37 spots were identified.

Only 26 proteins were characterized by MS (with fold-changes >2 and p < 0.05). Among these proteins, four involved in thiamine metabolism were distinctly represented in the HY and WT strains. A probable sulfur carrier protein, ThiS (Spot 43), was identified. ThiS has been reported to form a ThiS-ThiF complex, a key component of the sulfur transfer system in thiazole formation [35,36]. In addition, a probable thiazole biosynthetic enzyme (spot 285) that might be involved in thiazole biosynthesis [31,37] was 2.1-fold over-expressed in the HY strain compared to the WT strain. Thiazole and pyrimidine moieties are the construction units for thiamine, a precursor of TPP. TPP is the cofactor for 20 characterized enzymes involved in cellular bioenergetics processes leading to ATP synthesis [20]. TPP is also essential for the biological activities of pyruvate dehydrogenase (involved in pryruvate decarboxylation), α-ketoglutarate (TCA cycle) and transketolase (pentose phosphate pathway). Increasing the TPP levels would favor the accumulation of valine, a precursor amino acid for CPC biosynthesis [15].

A probable pyruvate oxidase (spot 137) homotetramer was highly expressed in HY. Each subunit binds one FAD and one TPP in the presence of Mn^2+^ or Mg^2+^. In the presence of Mn^2+^, both the FAD and TPP coenzymes can form binary complexes with the apoenzyme, which are inactive in the native overall oxidation reaction [38]. Pyruvate oxidase converts the pyruvate to acetylphosphate and CO_2_, while TPP is indirectly activated by FAD that is mediated by the formation of the catalytically competent FAD-thiamine-pyruvate oxidase ternary complex [39]. FAD is a redox cofactor involved in several important reactions in metabolism [40]. FAD serves as a cofactor to acetyl-CoA dehydrogenase. FAD-dependent redox enzymes play indispensable roles in generating structural complexity during natural product biosynthesis [41]. In our comparative proteomics analysis, the FAD-dependent oxidoreductase superfamily (spot 29) was 2.32–fold overrepresented in the HY strain, which may promote ATP formation and contribute to enhanced CPC production.

The sulfur carrier protein ThiS, the thiazole biosynthetic enzyme, pyruvate oxidase and FAD-dependent oxidoreductase are all involved in TPP biosynthesis. Thiamine is the precursor of TPP, and the thiazole moiety is a component of thiamine. Thus, we concluded that *Acthi* is a thiazole biosynthetic gene.

First, it was necessary to show that the ActhiS protein is differentially expressed in the WT and HY strains. The Western blotting and RT-qPCR results corresponded with the comparative proteomics results. The *Acthi* gene sequence was derived from the RNA-seq results from *A. chrysogenum* ATCC 11550 in our lab and deposited into GenBank under accession number KF010923. The sequence is homologous with *THI4* from *S. cerevisiae* [42] and *sti35* from *Fusarium oxysporum* [43]. Later studies revealed that the *THI4* gene is involved in thiamine biosynthesis, in the pathway responsible for the production of the thiazole moiety. Its expression is induced by thiamine depletion [26]. Further study showed that *THI4* in *Saccharomyces* is a thiazole biosynthetic gene. *THI4*-bound metabolites might be adenylated, a process that was demonstrated by AMP production [23]. The function of *sti35* in *F. oxysporum* has a dual role in thiamine biosynthesis and general stress responses [44]. In our study, *Acthi* was not only involved in thiazole biosynthesis; it also affected conidia germination, amino acid metabolism and NADPH generation and eventually affected CPC production in *A. chrysogenum*.

To demonstrate that the *Acthi* gene is related to thiamine, the mutants obtained from the silencing and over-expression of the *Acthi* gene in the WT strain were incubated on MMC medium in the presence or absence of thiamine. The *Acthi*-silenced mutant grew poorly on MMC medium in the absence of thiamine. Similar results have been reported for *Fusarium oxysporum* [44] and *S. cerevisiae* [33]. Over the last few years, several structural and biochemical studies have provided insight into the unprecedented mechanisms of some proteins involved. Thiamin biosynthesis is particularly unusual in eukaryotes in that it cannibalizes essential cellular cofactors and relies on single turnover proteins, which succumb to enzymatic suicide [45]. In 2011, *S. cerevisiae* THI4p was reported to be involved in an iron-dependent sulfide transfer reaction. THI4p is a suicidal enzyme that undergoes only a single turnover event [46]. However, the mechanism by which *A. chrysogenum* ActhiS functions has not yet been characterized. In our study, the intracellular thiamine contents of the mutants were measured, and it was revealed that *Acthi*(−) had a lower thiamine content than WT. This finding could be the direct evidence that the *Acthi* gene is related to thiamine biosynthesis and is a thiazole biosynthesis enzyme.

Furthermore, the different expression levels of the *acthi* gene in the acthi mutants affected conidia germination, amino acid metabolism and NADPH generation and eventually affected CPC biosynthesis. In *A. chrysogenum*, arthrospores are yeast-like cells representing metabolically active cells. Their morphological differentiation into arthrospores coincides with the maximum production of CPC [12]. After 4 days of fermentation, the vegetative hyphae of the *Acthi-*silencing mutant swelled, but they did not form typical arthrospores. In the WT strain and the *Acthi*-overexpressed mutant, obvious arthrospores were observed, with a greater abundance in the mutant strain. The observed discrepancy in hyphal growth and arthrospore formation might explain the decrease and increase of CPC production in the mutants. It is known that the global regulators *AcveA*, *AscepH* and *CPCR1* control hyphal growth and arthrospore formation [4,10,47]. The transcriptional levels of these genes in the *Acthi*-silencing mutant were lower than in the WT strain. Therefore, we speculate that *Acthi* expression levels may affect CPC biosynthesis.

In addition, the *Acthi* gene might affect the expression of TPP-dependent enzymes and the intracellular concentrations of precursor amino acids and NADPH, which is involved in CPC biosynthesis. Transketolase, which connects the pentose phosphate pathway to glycolysis, feeds excess sugar phosphates into the main carbohydrate metabolic pathways to generate most of the NADPH required for cell growth [48]. TPP is the cofactor of transketolase. The expression changes of the *Acthi* gene in *Acthi*(−) and *Acthi*(+) affected the production of thiamine and TPP and then modified the transketolase activity to generate a corresponding NADPH. This result is consistent with the speculation that a high concentration of ribulose 5-phosphate (together with reducing power in the form of NADPH) is formed in the high-producing strain via the pentose phosphate pathway [15]. It is accepted that an increase in the NADPH levels strongly increases the β-lactam production [49-51]. Metabolic flux analysis indicated that the improvement of the CPC yield could be achieved through the optimization of NADPH and ATP generation [52]. Our results indicated that high concentrations of thiamine and TPP and a high transcription level of TK activate the pentose phosphate pathway to generate NADPH, which increases the CPC production.

The synthesis of cysteine for β-lactam biosynthesis is another crucial factor in β-lactam production. Cysteine biosynthesis begins with sulfide [53,54]. Sulfite is reduced to sulfide by the multi-enzyme complex sulfite reductase, which involves the transfer of six electrons from NADPH to the sulfur atom [55]. In addition, PDC catalyzes the oxidative decarboxylation of pyruvate to form acetyl-CoA and citrate, and TPP is a cofactor that can enhance this process. Acetyl-CoA is involved in cysteine biosynthesis and CPC biosynthesis (Figure 1). In our study, no significant changes in the PDC transcripts were seen in the *Acthi* mutants. However, the increased level of the TPP cofactor may activate the PDC enzyme and lead to elevated levels of acetyl-CoA. These results indicated that the *Acthi* gene affected the thiamine yield and consequently changed the cofactor TPP level, which altered the CPC production.

L-valine is another precursor for CPC biosynthesis. L-valine biosynthesis has four enzymatic steps with two moles of pyruvate as precursor metabolites [5]. As a cofactor, TPP regulates the activity of acetolactate synthase, the first rate-limiting enzyme in the transformation of pyruvate to form α-acetolactate (Figure 1). Thus, increasing the TPP level would favor valine accumulation. Our results showed that the ALS genes were also similar to the TK transcript changes, and the concentration of L-valine showed a 1.46-fold increase in *Acthi*(+) and a 0.635-fold decrease in *Acthi*(−) compared with the WT strain. Therefore, it seems likely that the *Acthi* gene affects valine biosynthesis, resulting in further changes in the CPC production.

Finally, it is likely that *Acthi* manipulation can cause differential CPC production in the mutants. Transcriptional analysis verified that the transcription levels of the CPC biosynthetic genes *pcbC*, *pcbAB*, *cefEF*, *cefG* decreased significantly during fermentation in the *Acthi* silencing mutant. However, *Acthi* over-expression in *A. chrysogenum* may trigger the up-regulation of CPC biosynthetic gene transcription in vivo. TPP is a sensing metabolite that can bind the riboswitch motif located on the non-coding region of the mRNA of thiamine biosynthetic genes [56]. However, how the TPP-binding riboswitch (THI-box) recognizes its ligand with high specificity and affinity remains unclear [57]. It is also unknown if Acthi over-expression or elevated TPP deregulates CPC biosynthesis-related gene transcription under a riboswitch mechanism. These questions require further investigation.

We have shown that the *Acthi* gene is related to the synthesis of thiazole and that its expression levels are positively correlated with CPC yield in the WT strain via the upregulation of CPC biosynthetic genes and arthrospore formation. Finally, we have demonstrated that the manipulation of *Acthi* expression enhanced CPC production in the HY strain, with more than a 10% increase in the total yield.

Recently, the total genomic sequence of *A. chrysogenum* was published [58], which will facilitate the study of other differentially expressed proteins identified from comparative proteomics between the HY and WT CPC producers. However, future work will still involve characterizing the function of an ORF-encoded protein and studying the means by which the related metabolic pathway affects CPC biosynthesis.

## Conclusions

The ActhiS protein was discovered from the comparative proteomic analysis of two *A. chrysogenum* strains and was sequenced by RNA-seq. We found that ActhiS is involved in thiamine biosynthesis. Overexpression and silencing of the *Acthi* gene affects the morphological differentiation and CPC production of *A. chrysogenum.* Mutants also displayed different intracellular contents in both the amino acid precursors of CPC biosynthesis and the transcription levels of CPC biosynthetic genes. *Acthi* could be a potential manipulating target for the molecular breeding of the CPC producer.

## Methods

### Strains and culture conditions

The HY strain, *A. chrysogenum* 84-3-81-41, (in which the CPC production is higher than 15 mg mL^−1^) and the WT strain, *A. chrysogenum* ATCC 11550 (in which the CPC production is lower than 4 mg mL^−1^) were used in this study. These strains were grown on agar medium [3]. The procedures of fermentation and the detection of CPC production were performed as described previously [34]. MMC [59] was used for the growth analysis of the *A. chrysogenum* mutants. *A. chrysogenum* and its mutants were grown on thiamine-supplemented medium (1 μg mL^−1^ thiamine + MMC medium) at 28°C for 7 days. All plasmids and oligonucleotides used in this work are shown in Tables 2 and 3.

**Table 2:** **Oligonucleotide primers used in this work**

***Primers for RT-qPCR***	
5’-*Acthi*-qPCR	5’-CATGGGTGCCTTCTCCGTCA-3’
3’-*Acthi*-qPCR	5’-GACCATGGCACCAAAGGTAGGT-3’
5’-*AcveA*	5’-GTTCGAGGAGGGCAAGGAAATCA-3’
3’-*AcveA*	5’-GGTCAAGATGGGAGGGTTGGGTT-3’
5’-*AcsepH*	5’-GACAAAGAAGCGCAGGTGGTGG-3’
3’- *AcsepH*	5’-GGGTTTACCTTGGAGCAGCTCGATA-3’
5’-*cpcR1*	5’-TGACGTCAAAGGAGCCAGTCGT-3’
3’-*cpcR1*	5’-GCGGCTGCGGAACAATGTAA-3’
5’-*pcbAB*	5’-CGCTTGGCCGAGGAGAGAAA-3’
3’-*pcbAB*	5’-ATGCATGCATCCAGCTGGCC-3’
5’-*pcbC*	5’-AGGACATCCAGGCTGACGACACG-3’
3’-*pcbC*	5’-CGCTCCTCGTTGACCCATTTGA-3’
5’-*cefEF*	5’-CGCCGTTCTCAACTCTGTGGGC-3’
3’-*cefEF*	5’-CGAGCGTGATGGTCGATAGGTCGTAG-3’
5’-*cefG*	5’-GTTCGTATTCATCGCCAGGT-3’
3’-*cefG*	5’-TATTCCGGCCGGCTTGGACT-3’
5’-*AHAS*	5’-ATGCCACGTACAACGACATCGC-3’
3’-*AHAS*	5’-CTTTGCGGCGTTGAACTCGG-3’
*5’-PDC*	5’-TTGAGGATGTGACGCTTCGGG-3’
*3’-PDC*	5’-TTGCCTACAAAGCCCTCCGC-3’
5’*-TK*	5’- ATGGGCTACGGCGAGATTGACC-3’
3’*-TK*	5’- TCCAGAGGACGTGGGCAATG-3’
5’actin-qPCR	5’-GCCGCCCTCGTTATCGACAA -3’
3’actin-qPCR	5’- CGTACGAGTCCTTCTGGCCCAT -3’
*Primers for Acthi gene cloning and the construction of the pYG237 and pYG239 plasmids*	
*Acthi*-R1	GCG***GAATTC***ATGTCGCCTCCCGCTGCTAC
*Acthi-*R2	CCG***TCTAGA***TCAGACCGCATTCTCCTTCT
*Acthi*-sense-R3	CG***GAATTC***GGCTTGAGCACCGCCTAC
*Acthi*-sense-R4	GGGAATTC***CATATG***GTTGGGGTCCATGCAGGA
*Acthi*-antisense-R5	CG***TCTAGA***GGCTTGAGCACCGCCTAC
*Acthi*-antisense-R6	AT***CTCGAG***GTTGGGGTCCATGCAGGA
Intron-R7	TGCTCA***CATATG***TGAGCCTTTCTTCTTGCCTGTCA
Intron-R8	CG***CTCGAG***GTGTGTTTTGAACAATTTCACG

Letters in bold represent restriction endonuclease sites. R1: *Eco*RI, R2: *Xba*I, R3: *Eco*RI, R4: *Nde*I, R5: *Xba*I, R6: *Xho*I, R7: *Nde*I, R8: *Xho*I.

**Table 3:** **Plasmids used in this work**

**Plasmids**	**Genotype or description**	**Source**
pET28a	Vector for protein overproduction	Novagen
pET28a-*Acthi*	pET28a derivative for ActhiS over-expression from *E. coli*	This study
pYG013	Vector for gene expression *in A. chrysogenum*	Zhang (2004) [60]
pYG237	pYG013 derivative for *Acthi* over-expression in *A. chrysogenum*	This study
pYG239	pYG013 derivative for silencing *Acthi* in *A. chrysogenum*	This study

### 2-DE separation

Intracellular proteins were harvested from the mycelium of *A. chrysogenum* in the fermentation stage [60]. One-milligram protein samples were mixed with a thiourea rehydration solution (8 M urea, 2% CHAPS, 0.3% IPG buffer, 10 mM DTT, 0.002% bromophenol blue). A total of 450 μl of sample solution was loaded onto ReadyStrip Linear IPG strips (pH3-10 non-linear [NL], 24 cm; GE) at room temperature for 2 h (rehydration). Then, the strips were loaded onto an Ettan™ IPGphor™ 3 IEF System (GE). Proteins were focused at 20°C using the following program: 100 V, 3 h; 500 V, 2 h; 1000 V, 2 h; 3000 V, gradient to 2 h; 8,000 V, gradient to 8 h; 10,000 V, 5 h; 500 V, 5 h. The focused IPG strips were first equilibrated for 15 min with equilibrium bufferI(6 M urea, 50 mM Tris–HCl pH 8.8, 2% w/v SDS, 1% w/v DTT, 0.002% bromophenol blue). For the second equilibration, equilibrium bufferII (DTT was replaced with 2.5% iodoacetamide for the equilibrium buffer) was used. For the second-dimension polyacrylamide gel electrophoresis (SDS-PAGE), the equilibrium strips were placed onto 12.5% polyacrylamide in an Ettan Dalt Six apparatus (GE Healthcare) for 1 hour at 1watts/gel and then 2 watts/gel for 10 hours. Proteins were stained with Coomassie Blue G-250 (Amersco) for at least 3 h and destained in 10% (v/v) acetic acid until a clear background was obtained.

### Protein expression level analysis

Three different gels from each sample were prepared. Two-dimensional images were captured by scanning the stained gels using Image Master 2D Platinum 7.0 software (GE) to detect spots, match gels, and analyze interclass relationships. To ensure a high level of reproducibility of spot volumes in the gels produced in triplicate, the spots were detected in at least two of the triplicates. Automatically detected spots were checked manually to eliminate artifacts due to gel distortion, abnormal staining or streaks. After background subtraction, normalization and matching, spot normalization was completed using relative volumes to quantify and compare the gel spots. Relative volumes corresponded to the volume of each spot divided by the total volume of all of the spots in the gels. For analysis of protein expression between the HY and WT strains, a protein was considered to be differentially expressed when the changes in the normalized spot intensities were at least 2-fold, with a statistical significance level of p < 0.05 (Student’s t-test).

### Mass spectrometric analysis and database searching

The differentially expressed protein spots were manually excised from the Coomassie Blue G-250-stained gels and were then in-gel digested with trypsin. First, the gel pieces were destained three times using a fresh solution of 15 mM potassium ferricyanide and 50 mM sodium thiosulfate (1:1). Then, the gel pieces were washed twice in 100% acetonitrile and re-hydrated on ice using a solution of sequencing grade modified trypsin (Promega) in 20 mM ammonium bicarbonate. Proteins were digested at 37°C for more than 12 h at a trypsin concentration of 12.5 ng/μl. After digestion, peptides were analyzed in a 4700 MALDI-TOF/TOF Proteomics Analyzer (Applied Biosystems) according to the manufacturer’s instructions. Combined MS and MS/MS spectra were submitted to MASCOT (V2.1, Matrix Science) using GPS Explorer software (V3.6, Applied Biosystems) and were searched in the NCBI database (release date: 2009) for fungal taxonomy. MASCOT protein scores (based on the MS and MS/MS spectra) greater than 72 were considered to be statistically significant (p < 0.05). The individual MS/MS spectrum with statistical significance (confidence interval >95%) and the best ion score (based on the MS/MS spectra) was accepted. To eliminate the redundancy of proteins that appeared in the database under different names and accession numbers, the protein belonging to the species *A. chrysogenum* or with the highest protein score (top rank) was singled out.

### Expression and purification of ActhiS

The total RNA of the WT strain has been sequenced [34]. The sequence of the *Acthi* gene was deposited into GenBank under accession number KF010923. The *Acthi* gene encoding the *A. chrysogenum* thiazole biosynthesis enzyme was amplified with primers R1 and R2 (Table 2). To over-express *Acthi* in *E. coli*, the amplified DNA fragment containing the coding region of *Acthi* was digested with *EcoR*I and *Hin*dIII and was then inserted into the corresponding sites of pET28a to generate the recombinant plasmid pET28a-*Acthi*. The recombinant plasmids were verified by sequencing and were subsequently introduced into *E. coli* BL21 (DE3) for protein expression. ActhiS was over-expressed and purified as a His_6_-tagged fusion protein using Ni-NTA agarose chromatography according to the manufacturer’s protocol (Novagen). The concentration of total protein was determined using a Bradford Protein Assay Kit (Sangon Biotech) with bovine serum albumin (BSA) as the standard. Then, the purified protein was stored at −70°C.

### Preparation of antibodies and Western blotting

Approximately 4 mg of the purified ActhiS with Freund’s complete adjuvant was injected into one healthy rabbit four times (once per week). Antisera were collected from the rabbits after one month and were used as polyclonal antibodies against the ActhiS protein.

To examine the expression of *Acthi* in *A. chrysogenum* during fermentation, the HY strain, the WT strain, *Acthi*(−), *Acthi*(+) and the HY-*Acthi*(+) strains were inoculated into fermentation medium as described previously. The fermentation was conducted for 4 and 7 days. Mycelia were harvested for detecting *Acthi* expression through Western blotting. The protein extraction protocol used is described in Liu’s methods [60]. The concentration of total protein was determined using the Bradford Protein Assay Kit (Sangon Biotech) using BSA as the standard. Equal amounts of crude protein extract (15 μg) samples were loaded onto 12% SDS-PAGE. Proteins in the gels were transferred to polyvinylidene difluoride (PVDF) membranes (Millipore) and incubated with anti-ActhiS antibody (1:500 dilution) and anti β-actin antibody. The horseradish peroxidase (HRP)-conjugated secondary antibody (Abmart, Shanghai, China) was used at a dilution of 1:5,000, followed by detection with a SuperSignal West Pico Mouse IgG Detection Kit (Pierce USA).

### RNA isolation and RT-quantitative PCR

Total RNA was isolated using TRIzol Reagent (Sangon, Shanghai) according to the manufacturer’s instructions. The RNA samples were treated with RNase-free DNase (Takara) to remove genomic DNA. The PrimeScript™ RT reagent Kit (Takara) was used for the reverse transcription. Synthesis of cDNA and RT-qPCR were performed as described previously [34].

### Construction of the Acthi over-expression vector and the silencing vector and *A. chrysogenum* transformation

The *Acthi* gene encoding the *A. chrysogenum* thiazole biosynthesis enzyme was amplified with primers R1 and R2 and expressed under the control of the *pcbAB* promoter [3] in pYG237 (Figure 4A). Two 402-bp DNA sense and antisense fragments were cloned from the cDNA with primers R3 and R4 and R5 and R6. The 147-bp intron sequence was amplified from pSilent-1 [61] using primers R7 and R8 (Figure 4A). The three fragments were ligated into the plasmid pYG013 [62] to construct plasmid pYG239 (Figure 4B). Subsequently, the recombinant plasmids pYG237 and pYG239 were introduced into the *A. chrysogenum* WT strain as described previously [63]. ActhiS over-expression and silencing mutants were selected based on the phleomycin-resistant phenotype at 5 μg mL^−1^.

### Microscopy and image analysis

Morphological differentiation of the *A. chrysogenum* strains was observed under a scanning electron microscope (Nikon Eclipse E200).

### Detection of thiamine in *A. chrysogenum* and Acthi mutants

The mycelia of *A. chrysogenum* after fermentation were harvested by centrifugation and washed with sterilized distilled water three times. Samples were then freeze-dried, and 10 mg mycelium was supplemented with 10 mL of a 0.1 M HCl solution. After ultrasonication, the supernatant was placed in the autoclave at 121°C for 30 min of hydrolysis. The resulting supernatant was collected after centrifugation at 13,400 g for 25 min at 4°C. An equal volume of 10% trichloroacetic acid (TCA) was added to the supernatant to precipitate the protein component. The precipitated sample was thoroughly vortex-mixed and centrifuged for 10 min. The final supernatant was used for the subsequent thiamine extract analysis.

Thiamine was measured with a microplate fluorometer reader (BioTek-Synergy 2). This method was adapted from that described in Li’s report [64]. The thiamine was derived from alkaline potassium ferricyanide (4 mL 0.1% potassium ferricyanide mixed with 60 mL 15% NaOH) to thiochromes. Eighty milliliters of thiamine standard working solutions (0.1-1 μg mL^−1^ thiamine standard) was mixed with 50 μL alkaline potassium ferricyanide or 50 μL 15% NaOH to load the 96-well plate by mild shocking for 30 s, followed by the addition of 20 μL methanol. The plate was then placed into a microplate reader to measure the fluorescence intensity of each well at a 375 nm excitation wavelength and a 435 nm emission wavelength. The standard curve was calculated based on the results (data not shown). The thiamine concentrations in the extract samples were measured using the same protocol according to the standard curve.

### Sample preparation for the determination of intracellular metabolites

Aliquots of 10 mg freeze-dried mycelium from *A. chrysogenum* fermentation, 100 μL methanol-chloroform-ddH_2_O (3:1:1 v/v) and L-2-chlorophenylalanine (in 5 μL of water and methanol) were mixed for metabolite extraction at 70 Hz for 300 s on a tissuelyzer. After centrifugation at 12,000 g for 10 min, the supernatant was dried completely in a vacuum concentrator. An aliquot of 80 μL methoxyamine hydrochloride (15 mg mL^−1^ in pyridine) was added to the residue and incubated at 37°C for 90 min for methoxyamination. The sample was then trimethylsilylated by adding 80 μL bis-trimethylsilyl trifluoroacetamide (with 1% Trimethylchlorosilane) and then incubated at 70°C for 60 min. The derived samples were cooled to room temperature before analysis.

### GC/MS analysis of amino acids

All samples were analyzed in a 7890A gas chromatograph coupled with a 5975C mass spectrometer (Agilent Inc., CA, USA). An ADB-5 ms capillary column (30 m × 250 μm inner diameter, 0.25 μm film thickness; J&W Scientific, Folsom, CA, USA) was used to separate the compounds. The injector port was heated to 270°C, and injections (1 μL) were performed with a split ratio of 5:1. Helium (purity > 99.999%) was used as the carrier gas at a constant flow of approximately 1 mL/min. The column temperature was heated to 60°C for 1 min, then increased to 300°C at 5°C/min, and held for 16 min. The total run time was 65 min. The temperatures of the transfer line, ion source and quadruple were maintained at 280, 230 and 150°C, respectively. Electron impact ionization mass spectra were recorded with an ionization energy of 70 eV and an EM voltage of 1718 V. Mass spectra were scanned from 33 to 600 amu in total ion chromatogram (TIC) mode after a solvent delay of 6.5 min. The metabolite identification was performed by the National Institute of Standards and Technology (NIST) mass spectral library (2011) in MSD ChemStation (version E.02.02.1431; Agilent Inc., CA, USA). L-2-chlorophenylalanine was also utilized to assess the process variability during sample preparation and data processing.

## Abbreviations

CPC: Cephalosporin C
HY: High-yield CPC producer
WT: Wild-type strain
ActhiS: Thiazole biosynthesis enzyme
2-DE: Two-dimensional gel electrophoresis
GC-MS: Gas chromatography mass spectrometry
TPP: Thiamine diphosphate
FAD: Flavin adenine dinucleotide
MMC: Chemically defined medium
ALS: Acetolactate synthase
TK: Transketolase
PDC: Pyruvate decarboxylase
KGDH: α-ketoglutarate dehydrogenase
*pcbAB*: L-α-aminodipyl-L-cysteinyl-D-valine synthetase gene
*pcbC*: The isopenicillin N-synthetase gene
*cefEF*: The bifunctional deacetoxycephalosporin C synthase/hydroxylase gene
*cefG*: DAC acetyltranferase gene
